# Observations on the Use of Quinine and Opium, in Acute Rheumatism

**Published:** 1850-07

**Authors:** 


*Observations on the use of Quinine and Opium, in Acute Rheumatism.* By JOSEPH PARRISH, M. D., Editor of the New Jersey Medical Reporter.

Being some miles from home, waiting the movements of an obstetric patient, I conclude to fill some of the blank sheets of my memorandum book, for the printer, with observations on the use of quinine and opium in acute rheumatism. Almost every physician with ample opportunities for experience, after a few years practice, selects from the materia medica his favorite remedies for the treatment of certain diseases; for though we cannot always trace in the mind, a clear adaptation of the known properties of a medicine, to the morbid condition of the system for which we may be called upon to prescribe, we are sometimes tempted to depart from the beaten track, and enter the field of hypothesis, when we feel assured by past experience, that there is room for improvement. Acute rheumatism is a very common disease, for which a common mode of treatment has long been acknowledged. The presence of severe pain, active external inflammation, and a pulse above the normal standard, would seem to indicate the propriety of blood-letting as the primary remedy; such is the received opinion of the profession, and it is believed the majority of practitioners act in accordance with it. My own experience has not warranted the adoption of this opinion. The symptoms of the disease under consideration need not be recapitulated here, they are familiar to the whole profession; but there is one peculiarity in rheumatic inflammation which deserves special notice as having an important bearing upon the treatment; it is its fugitive character, its tendency to remove suddenly from place to place, and scarcely ever to terminate in suppuration. At any rate it may be called a *peculiar* inflammation.

If a patient complains of acute throbbing pain in any part of the body, and the skin becomes inflamed and hot, pressure intolerable, or even the slightest touch, and we witness the progress of this action from day to day, till suppuration is established, and an abscess forms, we see a very different state of things from what happens in rheumatic pain and fever. In the latter the suffering may be as acute, the local inflammation as decided, and yet it will suddenly leave that part, and as suddenly fix itself upon a distant region of the body, or perhaps, if there exists any irritation or functional derangement of any of the internal viscera, it may be located there, as in the heart, stomach, or other organ. We have considered this to be a distinctive peculiarity of rheumatic inflammation, which demands of the practitioner a mode of treatment different from what is applicable to common inflammation. Acting upon this view of the subject, the disease has recently been treated as follows, with the happiest results: To evacuate the elementary canal as a preliminary step, is obviously important; for this purpose I generally prescribe a combination of ten grains each, of blue mass and prepared chalk, to be followed in a few hours with a full dose of magnesia and wine of colchicum; the bowels being freely moved, from five to ten grains of quinine, with from one to two grains of opium, according to the age and constitution of the patient, and the severity of the pain, are given at night; this will generally procure sleep and freedom from suffering during the following day; the effervescing mixture or small doses of Dover's powder are continued at intervals of two or three hours, and bland mucilaginous drink allowed, which serve to nourish the patient, and keep the bowels soluble. I have not unfrequently known the combination of quinine and opium to operate as a laxative, and thus remove the necessity of any other cathartic. Some twenty cases of the disease I have treated on this plan; not one of them lost a drop of blood, and they all recovered speedily; most of them were females.

The applicability of quinine in scrofulous inflammation is generally admitted; in rheumatic ophthalmia it is often employed, and in erysipelas its efficiency is not questioned. As a journalist and a practitioner of medicine, we are not at liberty to withhold from the common stock, any thing that promises advantage to the sick, or to the profession; hence, these views are submitted with due deference to the opinions of those of more advanced age and experience, and who have enjoyed greater opportunities of treating the disease; but they are the result of careful observation, within the limited sphere of my professional labors, and as such, are submitted to the medical public.—*New Jersy Medical Reporter.*

